# Necrostatin-1 Reduces Neurovascular Injury after Intracerebral Hemorrhage

**DOI:** 10.1155/2014/495817

**Published:** 2014-03-06

**Authors:** Melanie D. King, Wittstatt A. Whitaker-Lea, James M. Campbell, Cargill H. Alleyne, Krishnan M. Dhandapani

**Affiliations:** Department of Neurosurgery, Medical College of Georgia, Georgia Regents University, 1120 15th Street, Augusta, GA 30912, USA

## Abstract

Intracerebral hemorrhage (ICH) is the most common form of hemorrhagic stroke, accounting for 15% of all strokes. ICH has the highest acute mortality and the worst long-term prognosis of all stroke subtypes. Unfortunately, the dearth of clinically effective treatment options makes ICH the least treatable form of stroke, emphasizing the need for novel therapeutic targets. Recent work by our laboratory identified a novel role for the necroptosis inhibitor, necrostatin-1, in limiting neurovascular injury in tissue culture models of hemorrhagic injury. In the present study, we tested the hypothesis that necrostatin-1 reduces neurovascular injury after collagenase-induced ICH in mice. Necrostatin-1 significantly reduced hematoma volume by 54% at 72 h after-ICH, as compared to either sham-injured mice or mice administered an inactive, structural analogue of necrostatin-1. Necrostatin-1 also limited cell death by 48%, reduced blood-brain barrier opening by 51%, attenuated edema development to sham levels, and improved neurobehavioral outcomes after ICH. These data suggest a potential clinical utility for necrostatin-1 and/or novel necroptosis inhibitors as an adjunct therapy to reduce neurological injury and improve patient outcomes after ICH.

## 1. Introduction

Spontaneous intracerebral hemorrhage (ICH) accounts for ~15% of all strokes and induces a 30-day mortality rate of ~40% [1–5]. The rupture of small vessels damaged by chronic hypertension or amyloid angiopathy induces primary ICH, creating a space-occupying hematoma within the brain parenchyma. Hematoma volume directly correlates with neurological deterioration and patient mortality [2, 6–9] and neurosurgical clot evacuation produces more favorable outcomes in subsets of ICH patients; however, many patients are not amenable to surgical intervention due to hematoma location or concurrent intraventricular hemorrhage [10]. As such, conservative management remains a clinical mainstay, reinforcing the notion that ICH is the least treatable form of strokes and stressing the need for novel therapeutic approaches.

Vascular injury and inflammatory activation are predictive markers of hematoma enlargement, development of vasogenic edema, and acute neurological deterioration [11, 12]. Apoptosis of cerebral endothelial cells increased blood-brain barrier (BBB) permeability, vasogenic edema, and neurological impairment after hemorrhagic stroke [13, 14]. Furthermore, activation of the proinflammatory transcription factor, NF*κ*B, correlated with apoptotic cell death within perihematomal blood vessels after ICH [15]. Thus, a reduction in neurovascular injury may improve outcomes after ICH; however, the underlying mechanisms remain poorly defined and contribute to the lack of medical treatment options after ICH.

Inflammation is a conserved immune response to tissue injury. Hemolysis of extravasated erythrocytes triggers the release of proinflammatory mediators in and around the hematoma core. Along these lines, inflammatory activation correlates with increased hematoma expansion, neurological deterioration, and a poor functional recovery after ICH [12, 15–17]. Notably, elevated plasma concentrations of the proinflammatory mediator, tumor necrosis factor-*α* (TNF-*α*), clinically correlated with acute hematoma enlargement, edema development, and patient outcome following ICH [18–22]. Similarly, TNF-*α* expression was acutely increased within the perihematoma tissue using multiple species and models of experimental ICH [23–28]. Coupled with our finding that neurovascular injury directly correlated with TNF-*α* expression after ICH in mice, TNF-*α* may induce acute neurological injury after ICH; however, the mechanisms underlying TNF-*α*-induced neurovascular injury after ICH remain poorly defined.

Recent evidence suggests that TNF-*α* induces necroptosis, a novel form of cell death that exhibits features of apoptosis, necrosis, and type 2 autophagic death [29–32]. Although the role of necroptotic cell death after ICH remains unexplored, we first reported that hemin, a hemoglobin oxidation byproduct that accumulates within intracranial hematomas [33], induced TNF-*α* expression and promoted necroptotic cell death in cultured astrocytes [34]. Receptor interacting protein kinase 1 (RIPK1) is a multifunctional protein kinase that interacts with TNF-*α* receptor (TNFR) to promote NF*κ*B and to activate necroptotic cell death [35–37]. Thus, RIPK1 may represent a novel therapeutic target after ICH. Herein, we hypothesized that necrostatin-1 (Nec-1), a novel and highly selective RIPK1 inhibitor, improves neurological outcomes following ICH.

## 2. Materials and Methods

### 2.1. ICH Model

**Figure 1:**
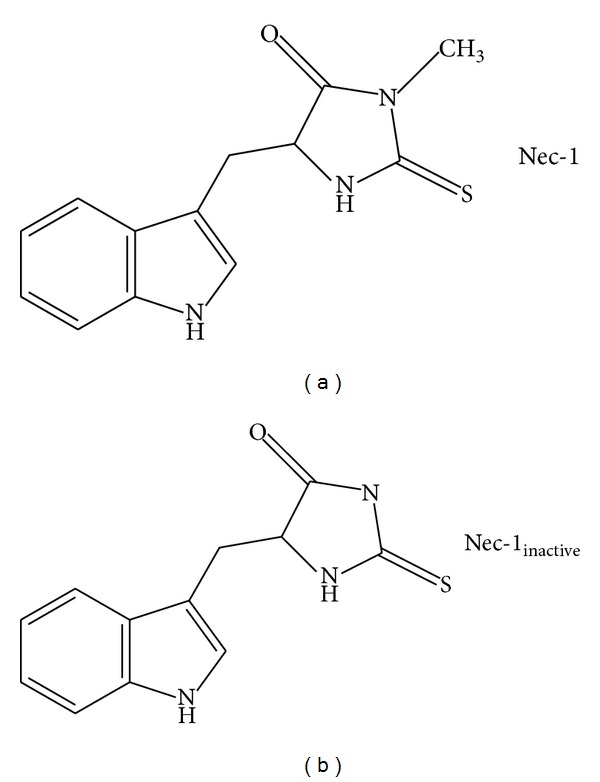
Chemical structures of Nec-1 and Nec-1_inactive_.

Animal studies were reviewed and approved by the Committee on Animal Use for Research and Education at Georgia Regents University (Protocol number 2008-0166), in compliance with NIH guidelines. Male CD-1 mice (8–10 weeks old; Charles River, Wilmington, MA, USA) were anesthetized with a cocktail of 8 mg/kg xylazine and 60 mg/kg ketamine. Throughout all surgical procedures, body temperature was maintained at 37°C by using a small-animal temperature controller (David Kopf Instruments, Tujunga, CA, USA). Mice were placed into a stereotactic frame and a 0.5 mm diameter burr hole was drilled over the parietal cortex, 2.2 mm lateral to the bregma. A 26-gauge Hamilton syringe, loaded with 0.04 U of bacterial type IV collagenase in 0.5 *μ*L saline, was lowered 3 mm deep from the skull surface directly into the left striatum. The syringe was depressed at a rate of 450 nL/min and left in place for 10 minutes after the procedure to prevent solution reflux and excess diffusion. Sham animals underwent the same surgical procedure, except that saline was stereotactically injected rather than collagenase. After the syringe was removed, bone wax was used to close the burr hole, the incision was surgically stapled, and mice were kept warm until recovery of the righting reflex. This entire procedure was detailed previously by our structural analog (Nec-1_inactive_) (Tocris, Ballwin, MO, USA; see Figure 1), or saline (placebo) was administered via the intracerebroventricular (icv) route at the time of injury. This dose of Nec-1 and route of administration were based on previous studies showing efficacy in preclinical models of stroke and traumatic brain injury [37, 38].

### 2.2. Hematoma Volume

Hematoma volume was spectrophotometrically quantified by the QuantiChrom Hemoglobin Assay Kit (Bioassay Systems, Hayward, CA, USA), as per the manufacturer's recommendations and as routine to our laboratory [39]. The amount of hemoglobin in each hemisphere was calculated using the following: [(optical density of sample/optical density of calibrator)∗100].

### 2.3. BBB Permeability

BBB permeability was quantified following administration of Evans blue (20 mg/mL in PBS, i.v.) 2 h prior to sacrifice. Blood (100 *μ*L) was obtained by cardiac puncture and centrifuged, and then the plasma was diluted in N,N-Dimethylformamide (1 : 1000). Following perfusion with saline, brains were weighed, solubilized in N,N-Dimethylformamide, and then incubated at 78°C for 18 h. Absorbance was then determined in brain and blood samples at 620 nm using a Synergy HT plate reader (Bio-Tek, Winooski, VT, USA). The concentration of Evans blue (*μ*g/*μ*L) in each sample, a measure of BBB permeability, was calculated using a standard curve, and permeability was equal to [(Evans blue concentration of brain/weight of brain)/(Evans blue concentration of plasma/circulation time)], as reported previously by our group [39, 40].

### 2.4. Assessment of Cerebral Edema

Brain water content, an established measure of cerebral edema, was quantified in 2 mm coronal tissue sections of the ipsilateral or corresponding contralateral striatum, as routine to our laboratory [39–42]. Tissue was immediately weighed (wet weight) and then dehydrated at 65°C. Samples were reweighed 48 h later to obtain a dry weight. The percentage of water content in each sample was calculated as follows: % Brain water content = [((wet weight−dry weight)/wet weight)∗100].

### 2.5. RNA Isolation and qRT-PCR

Total RNA was isolated (SV RNA Isolation kit, Promega, Madison, WI, USA) and qRT-PCR was performed on a Cepheid SmartCycler II (Cepheid, Sunnyvale, CA, USA) using a Superscript III Platinum SYBR Green One-Step qRT-PCR kit (Invitrogen, Carlsbad, CA, USA), as per our laboratory [34, 39, 41–43]. Product specificity was confirmed by melting curve analysis and visualization of a single, appropriately sized band on a 2% agarose gel. Gene expression levels were quantified using a cDNA standard curve and data was normalized to RPS3, a housekeeping gene that is unaffected by the experimental manipulations. Data is expressed as fold change versus sham.

### 2.6. Immunohistochemistry

Deeply anesthetized mice were perfused with saline followed by 4% paraformaldehyde in PBS. Brains were removed and postfixed in 4% paraformaldehyde overnight at 4°C and then transferred into 30% sucrose. Tissue sections (20 *μ*m) were direct-mounted onto glass slides and stained using a primary antibody against glial fibrillary acidic protein (GFAP; Dako, 1 : 200), as detailed by our laboratory [41, 44]. After labeling with an Alexa-Fluor tagged secondary antibody, immunoreactivity was determined using a Zeiss LSM510 confocal microscope.

### 2.7. Propidium Iodide Staining

Propidium iodide (PI, 150 ng) was administered via tail vein five hours prior to sacrifice. Following tissue sectioning, brain sections were imaged by confocal microscopy. The number of PI positive cells was quantified by cell counts in three specified regions within the perihematomal regions. Counts were normalized to placebo-treated mice after ICH.

### 2.8. Neurological Outcomes

Neurological injury was determined using a modified 24-point scale, as detailed previously by our laboratory and others [39, 40, 45, 46]. This scale comprised six behavioral tests, each of which was graded from 0 (performs with no impairment) to 4 (severe impairment). A composite score was calculated as the sum of the grades on all six tests.

### 2.9. Statistical Analysis

One-way analysis of variance (ANOVA) followed by Student-Newman-Keuls or two-way ANOVA followed by Tukey's post hoc test was used for multiple group comparisons. Data are expressed as mean +/− SEM. A *P* value of <0.05 was considered to be significant.

## 3. Results

### 3.1. Necrostatin-1 Reduces Hematoma Volume after ICH

**Figure 2:**
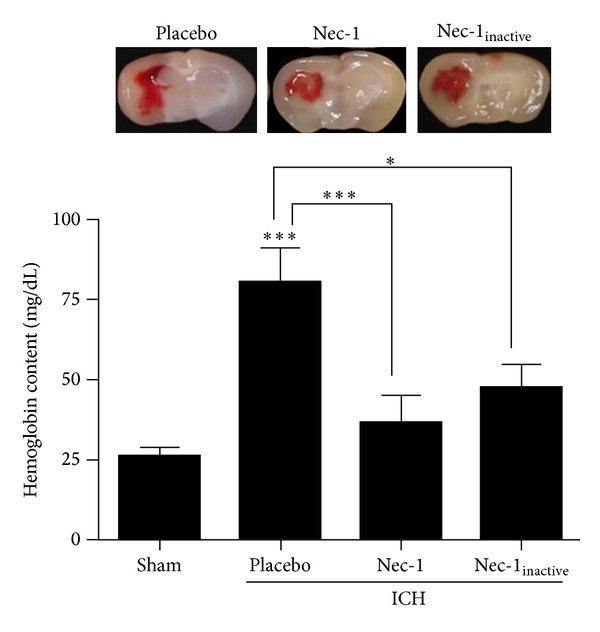
Nec-1 attenuates hematoma size after ICH. Nec-1 administration at the time of injury reduced hematoma size at 72 h after ICH. Coronal brain images were prepared and digitally captured to visualize hematoma size (top panels). Hematoma volume was quantified by determining the hemoglobin content of each hemisphere at 72 hours after ICH (bottom panel). Data are expressed as mean ± SEM (*P < 0.05, ***P < 0.001; *n* = 10–14 per group). Nec-1 and Nec-1_inactive_ were not significantly different from sham group.

Administration of Nec-1 significantly reduced hematoma volume within the ipsilateral hemisphere at 72 h after ICH (Figure 2). Specifically, hemoglobin content was increased within the injured hemisphere from 26.5 ± 2.6 mg/dL in sham-operated mice to 80.8 ± 9.9 mg/dL following ICH (*P* < 0.001 versus sham). Intrastriatal delivery of Nec-1 at the time of ICH reduced hemispheric hemoglobin content to 37.3 ± 8.1 mg/dL (*P* < 0.001 versus ICH, not significantly different from sham). Administration of Nec-1_inactive_ also reduced hemoglobin content after ICH (48.6 ± 7.2 mg/dL; *P* < 0.05 versus sham-, ICH-, and Nec-1-treated mice).

### 3.2. Cell Death Is Attenuated by Necrostatin-1 after ICH

**Figure 3:**
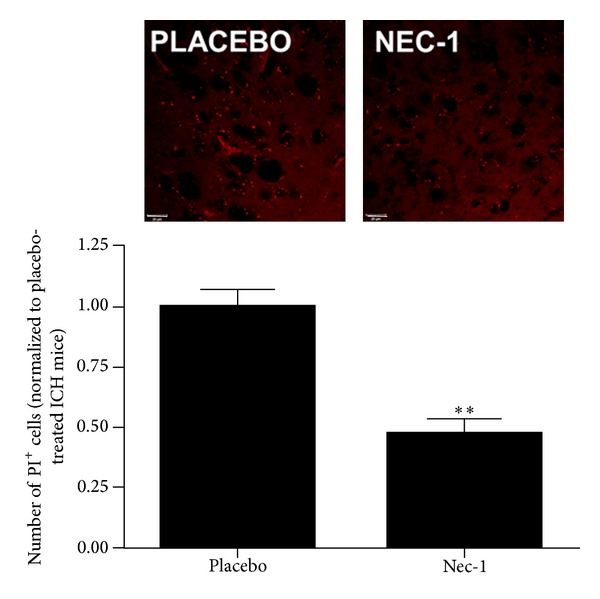
Nec-1 reduces cell death after ICH. Nec-1 administration at the time of ICH reduced perihematomal cell death, as assessed by PI staining. Top panels are representative images from placebo- or Nec-1-treated mice following ICH. Bottom panel depicts the quantification of PI staining following treatment with Nec-1 or Nec-1_inactive_. Cell counts were normalized to placebo-treated ICH mice. Data are expressed as mean ± SEM (**P < 0.01 versus placebo; *n* = 5 per group).

**Figure 4:**
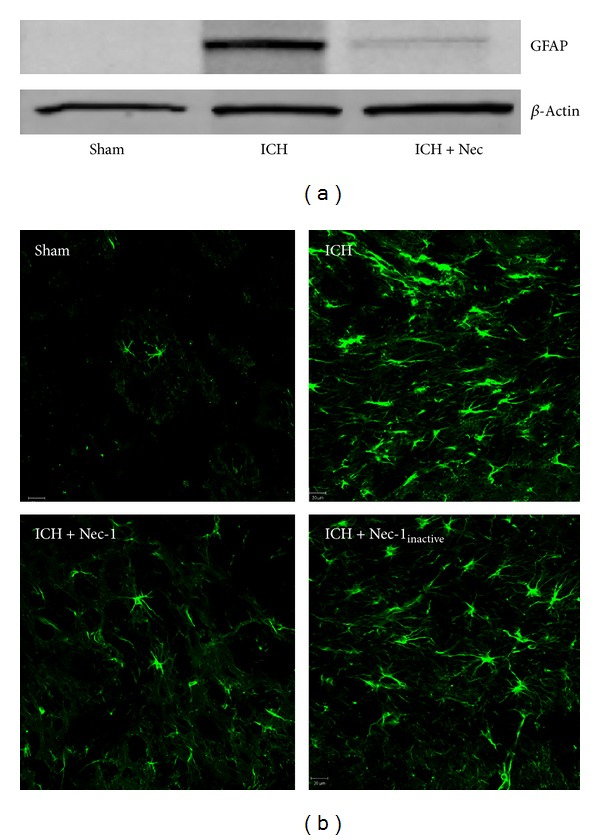
Effect of Nec-1 on ICH-induced reactive gliosis. Reactive astrogliosis was visualized by (a) Western blotting or (b) immunohistochemistry for glial fibrillary acidic protein (GFAP) at 72 h after ICH. GFAP immunoreactivity was increased and exhibited a characteristic stellate morphology after ICH. Administration of Nec-1 significantly reduced these effects whereas Nec-1_inactive_ was without effect. Data are representative of 5 mice per group. Scale bar = 20 *μ*m.

The effect of Nec-1 on hematoma volume was mirrored by brain lesion volume, as assessed by hematoxylin and eosin staining (data not shown). Consistent with this finding, Nec-1 reduced the number of propidium iodide positive (PI^+^) cells within the perihematomal tissue by 48%, as compared to ICH only mice (*P* < 0.01) (Figure 3). In contrast, Nec-1_inactive_ reduced the number of PI^+^ cells by 6.3% (not significantly different from placebo treated after ICH). Astrogliosis, a conserved response to brain injury [47, 48], was similarly reduced by Nec-1 treatment. Administration of Nec-1 reduced GFAP expression after ICH, as assessed by Western blotting and by immunohistochemistry, consistent with the attenuation of cellular injury (Figure 4). Coupled with our observation that Nec-1 attenuated proinflammatory gene expression, including reduced TNF-*α* expression (MEK and KMD, unpublished observations), these findings suggest that necroptosis contributes to glial reactivity after ICH.

### 3.3. Necrostatin-1 Reduces Neurovascular Injury after ICH

**Figure 5:**
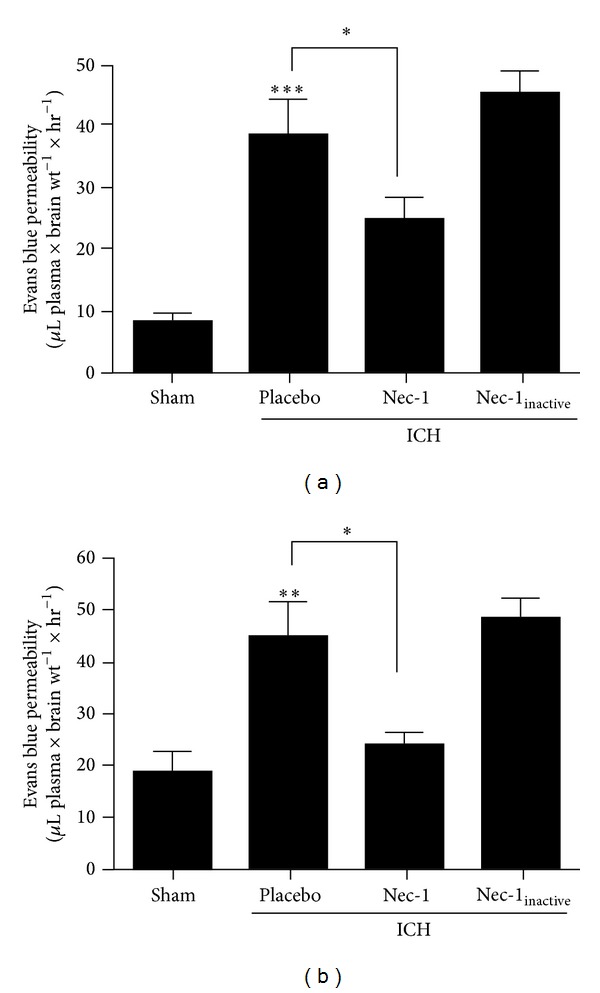
Nec-1 maintains blood-brain barrier integrity after ICH. Mice were administered Nec-1 or Nec-1_inactive_ at the time of collagenase-induced ICH. Evans blue extravasation, a sensitive measure of BBB disruption, was assessed at (a) 3 h or (b) 12 h after ICH. Data are expressed as mean ± SEM and were analyzed by one-way ANOVA followed by Student- Newman-Keuls post hoc test (**P < 0.01, ***P < 0.001, *n* = 7-8 per group).

Cerebral edema is a major cause of patient deterioration after ICH. Over the first 24 h after ICH, a significant increase in Evans blue extravasation, a sensitive measure of blood-brain barrier disruption, is observed. This breach in the blood-brain barrier was maximal between 3 and 12 h after injury, with spontaneous resealing observed at 24 h. Administration of Nec-1 at the time of injury significantly reduced Evans blue extravasation at all time points (Figure 5). In contrast, Nec-1_inactive_ did not significantly reduce blood-brain barrier opening at any time point, as compared to placebo-treated mice, suggesting a specific effect of Nec-1.

**Figure 6:**
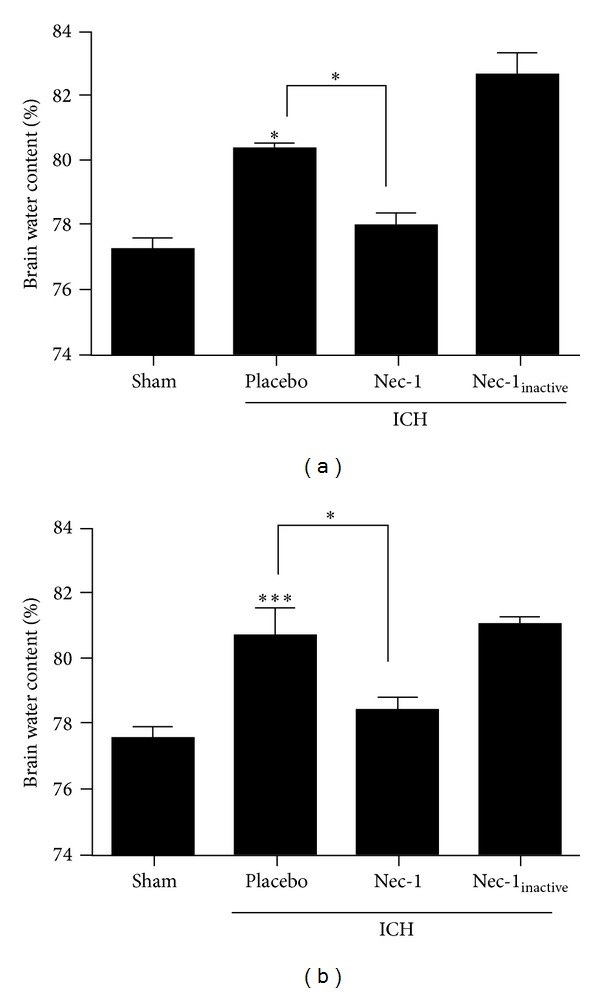
Nec-1 reduced edema development after ICH. Mice were administered Nec-1 or Nec-1_inactive_ at the time of collagenase-induced ICH. Brain water content, a measure of cerebral edema, was assessed in the ipsilateral hemisphere at (a) 24 h or (b) 72 h following ICH. Comparisons within each hemisphere between different treatment groups were done using a one-way ANOVA followed by Student-Newman-Keuls post hoc test (*P < 0.05, ***P < 0.001). Data are expressed as mean ± SEM from 10 mice per group.

BBB opening contributes to the development of vasogenic edema; thus, the effect of Nec-1 on edema development was next assessed. As was observed with Evans blue extravasation, Nec-1 reduced brain water content at all time points up to 5 days after ICH. The maximal effect of Nec-1 was noted at 24 h after ICH whereby brain water content was reduced from 80.3 ± 0.2% in placebo-treated ICH mice to 76.8 ± 0.4% following Nec-1 administration (*P* < 0.05 versus placebo) (Figure 6). As was observed with BBB opening, Nec-1_inactive_ was without effect on brain edema development after ICH (82.7 ± 0.7%; *P* < 0.05 versus Nec-1, not significantly different from placebo). No significant differences were observed between groups in the contralateral hemispheres.

### 3.4. Necrostatin-1 Improves Neurological Outcomes after ICH

**Figure 7:**
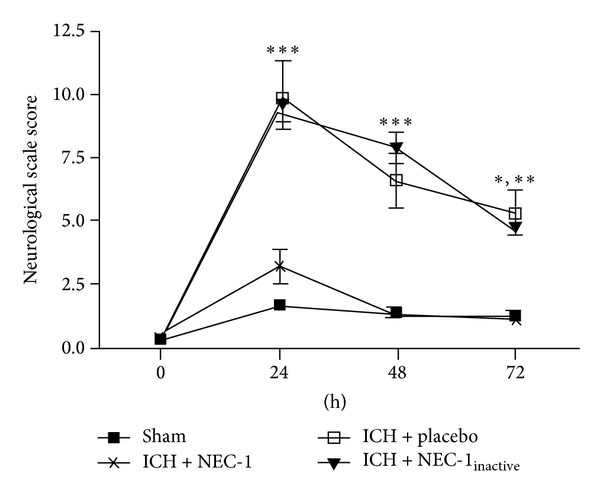
Nec-1 improves neurological outcome after ICH. Mice were administered Nec-1 or Nec-1_inactive_ at the time of collagenase-induced ICH. Neurological outcomes were assessed at 24 h, 48 h, or 72 h following sham or ICH. Data are expressed as mean ± SEM (*n* = 10/group) and were analyzed using a repeated measures ANOVA followed by Bonferroni's post hoc test (*P < 0.05, **P < 0.01, ***P < 0.001).

In line with a reduction in cell death and reduced edema development, necrostatin-1 improved neurological outcomes after ICH. Specifically, necrostatin-1 significantly improved the outcomes using a 24-point scale across the first 72 h after injury, as compared to either placebo- or Nec-1_inactive_-treated mice after ICH (*P* < 0.001 versus placebo-, Nec-1_inactive_-treated ICH mice) (Figure 7). Mice treated with necrostatin-1 were behaviorally not significantly different from sham-operated mice.

## 4. Discussion

ICH, the most common form of hemorrhagic stroke, is associated with the highest mortality and the worst long-term neurological outcomes of all stroke subtypes [6]. One-year mortality rates are >60% and of the ~67,000 Americans suffering an ICH annually, <20% recover functional independence after six months [1, 2, 49]. Notably, the incidence of ICH is expected to double over the next several decades due to an aging population and to changes in racial demographics [3]. These data emphasize the devastating nature of ICH and indicate the need for improved treatment options.

Extravasation of erythrocytes creates a space-occupying hematoma within the brain parenchyma [6–9, 50–54]. Hematoma growth continues over the ensuing hours due to rebleeding from the ruptured arteriole, bleeding in surrounding compressed vessels, and/or local clotting defects after vessel rupture [6, 8, 50]. The persistent or recurrent bleeding exacerbates the mass lesion, induces local compression of the microvasculature, and contributes to subsequent neurovascular dysfunction [3, 6–8, 50–53, 55, 56]. Hemolysis promotes spontaneous hematoma resolution; however, the concurrent production of hemoglobin degradation metabolites induces the release of proinflammatory mediators within and around the hematoma core. Along these lines, acute increases in TNF-*α* within the cerebrospinal fluid and plasma of spontaneous ICH patients correlated with patient mortality [22]. Furthermore, increased expression of FasL and a corresponding decrease in s-Fas (an inhibitor of Fas activation) were detected in perihematomal brain tissue from ICH patients, as compared to control patients [57]. Although the functional significance of the inflammatory response remains incompletely understood, acute expression of both FasL and TNF-*α* was associated with cellular injury and with edema formation after ICH [19, 57, 58]. These findings suggest a detrimental role for the early inflammatory response after ICH, yet the precise mechanisms whereby inflammation contributes to poor patient outcomes remain elusive.

Cell death is an important component of neurological injury after ICH, although the form(s) of cell death after ICH remain poorly defined. Features of both apoptotic and necrotic cell death appear within six hours of injury in perihematomal neurons and glia in preclinical ICH models and in postmortem human studies, with peak injury noted at three days [59–62]. Similarly, loss of plasmalemma integrity, a phenotypic hallmark of necrotic cell death, increased over the first three days after collagenase-induced ICH in mice [63], further suggesting a prominent role for necrosis after ICH. In contrast to the view of necrosis as a passive, irreversible form of cell death, necroptosis is a newly described form of programmed necrosis that is induced by FasL and/or TNF-*α* [64]. As activation of the proinflammatory transcription factor, NF*κ*B, was associated with cell death after ICH, we hypothesized that necroptosis may contribute to neurovascular injury after a brain hemorrhage. To test this possibility, we investigated whether Nec-1, a novel small molecule inhibitor of necroptosis [64], could reduce neurological injury after ICH.

Herein, we identified a novel role for Nec-1 in reducing cell death, attenuating hematoma expansion, limiting blood-brain barrier disruption, and restricting edema development after ICH. Our findings, which are in agreement with a recent report showing Nec-1 on apoptotic and autophagic cell death after collagenase-induced ICH [65], extend these findings using clinically relevant endpoints and suggest a potential role for targeting cell death pathways after ICH. Although the mechanism(s) whereby Nec-1 limited neurological injury were not explored in this study, it is notable that the biologically inactive, structural analog of Nec-1 did not exert the same protective effects of Nec-1 with respect to BBB opening, edema development, and behavioral outcomes; however, it is noteworthy that Nec-1_inactive_ attenuated hematoma volume to a similar magnitude as compared to Nec-1. Whereas these data suggest that the actions of Nec-1 may be selective rather than due to a nonspecific antioxidant effect, it remains unclear how Nec-1_inactive_ selectively exerted this effect on hematoma volume. Notably, a recent report suggested that Nec-1_inactive_ exerts biological activity on some necroptosis assays and sensitizes mice to lethality during systemic inflammatory response syndrome [66]. These interesting results suggest that caution should be taken in the use of Nec-1_inactive_ as a true biologically inactive control to Nec-1.

Receptor interacting protein 1 (RIP1), the proposed molecular target of Nec-1 [36], is a serine/threonine protein kinase implicated in NF*κ*B activation as well as in the initiation of necroptotic cell death [67]. Inflammatory activation at the time of admission was associated with early neurological deterioration in ICH patients [12] and NF*κ*B activation was sustained over the first several days in a preclinical ICH model [17]. We and others reported that NF*κ*B activation stimulates the expression of inflammatory mediators associated with necroptosis (e.g., TNF-*α*), induces blood-brain barrier permeability, increases edema development, and exacerbates neurobehavioral deficits after experimental ICH [15, 17, 39]. Furthermore, we reported that the NF*κ*B inhibitor, curcumin, promoted hematoma resolution and improved neurological outcomes after collagenase-induced ICH in mice [39]. Taken together, these results raise the unexplored possibility that RIP1 mediates acute neurological injury after ICH. Future work by our laboratory will characterize this interesting mechanism in further detail.

Nuclear blebbing and karyorrhexis were observed in glial cells within the white matter after intraventricular hemorrhage in preterm infants [68]. Astrocytic loss temporally preceded vascular injury after experimental ICH [28, 62] and focal astrocyte loss increased microvascular damage and induced transient BBB opening [69]. Although the cellular targets of Nec-1 after ICH were beyond the scope of the present study, these findings raise the possibility that the beneficial effects of Nec-1 observed herein may involve, at least in part, maintenance of glial function. Astrocytes are the primary source of the nonenzymatic antioxidant, glutathione, within the brain. We first reported that hemin rapidly depleted intracellular glutathione and induced caspase-independent cell death in murine astrocytes via an inflammatory mechanism [34]. Interestingly, this cellular injury was reversed by Nec-1, suggesting a role for necroptosis after hemorrhagic injury [34]. Our finding is consistent with a subsequent report demonstrating that glutathione depletion enhanced the release of neurotoxic substances, including TNF-*α* from human astrocytes [70]. Similarly, Nec-1 prevented glutamate-induced glutathione depletion and caspase-independent cytotoxicity in HT-22 cells [71]. Coupled with our recent finding that astrocyte-derived glutathione reduced hemorrhagic injury in cerebral microvessels [72], Nec-1 may improve neurological outcomes by limiting astrocytic dysfunction.

Necroptosis is a novel form of programmed cell death that is initiated by proinflammatory mediators, such as TNF-*α*. The model of ICH utilized in this study involves the intrastriatal injection of bacterial collagenase. Although this model best recapitulates the spontaneous intracerebral bleeding and evolving hematoma expansion observed in patients, collagenase may induce an exaggerated inflammatory response. Thus, the beneficial effects of Nec-1 may be overestimated. Nonetheless, cells exhibiting both apoptotic and/or necrotic phenotypes characteristic of necroptosis are observed in postmortem human brain sections, suggesting the validity of the protection observed in this study. Another limitation of the present study is that the mechanisms of Nec-1 protection remain undetermined. Although regarded as a highly specific RIP1 inhibitor [36], several* in vitro* studies suggest a possible direct antioxidant role for Nec-1. Furthermore, Nec-1 also may block indoleamine-2,3-dioxygenase (IDO), which catabolizes tryptophan into kynurenine. The inclusion of Nec-1_inactive_ is consistent with a selective effect of Nec-1 as this analogue does not reportedly exhibit antioxidant activity nor does it inhibit mouse IDO [73], although a recent conflicting report suggests that Nec-1_inactive_ may attenuate human IDO activity [66]. Thus, the possibility that some or all of the beneficial actions of Nec-1 are mediated via RIP1-independent mechanisms after ICH cannot be excluded. Future work by our laboratory will further characterize the specific therapeutic role of RIP1 targeting after brain hemorrhage. Regardless of the precise cellular mechanism, these studies suggest a novel protective role for Nec-1 after ICH.

## 5. Conclusions

The necroptosis inhibitor, Nec-1, reduced neurovascular injury and improved outcomes when administered at the time of injury in a preclinical model of ICH. These data suggest that therapeutic targeting of programmed necrosis may improve patient outcomes after a brain hemorrhage. Future work with more selective necroptosis inhibitors will establish the therapeutic window for improving neurovascular outcomes after ICH, providing a framework for potential clinical translation of these findings.
